# Liquid biopsy in breast cancer: current biomarker platforms and clinical applications

**DOI:** 10.37349/etat.2026.1002394

**Published:** 2026-08-05

**Authors:** Pritam Saha Podder, Debasree Bhadra, Canio Martinelli, Salvatore Cortellino, Antonio Giordano, Stefano Cinti

**Affiliations:** N.N. Petrov Research Institute of Oncology, Russian Federation; ^1^Department of Pharmacy, Jahangirnagar University, Dhaka 1342, Bangladesh; ^2^Pharmacy Discipline, Khulna University, Khulna 9208, Bangladesh; ^3^Sbarro Institute for Cancer Research and Molecular Medicine, Center for Biotechnology, College of Science and Technology, Temple University, Philadelphia, PA 19122, USA; ^4^Scuola Superiore Meridionale, 80134 Naples, Italy; ^5^Department of Medical Biotechnologies, University of Siena, 53100 Siena, Italy; ^6^Department of Pharmacy, University of Naples Federico II, 80138 Naples, Italy

**Keywords:** liquid biopsy, breast cancer, circulating tumor DNA, extracellular vesicles, microRNA, circulating tumor cells, precision medicine, biosensor

## Abstract

Breast cancer remains one of the leading causes of cancer-related mortality worldwide, and despite considerable advances in therapeutic strategies, the absence of reliable, minimally invasive diagnostic tools continues to limit early detection and real-time disease monitoring. Liquid biopsy has emerged as a transformative paradigm in oncology, offering the capacity to integrate tumor biology through the analysis of tumor-derived materials that circulate in peripheral blood and other biofluids. This review provides a comprehensive overview of the key analytes employed in liquid biopsy, including circulating tumor DNA (ctDNA), circulating tumor cells (CTCs), microRNAs (miRNAs), extracellular vesicles (EVs), and protein biomarkers with particular emphasis on their translational relevance in breast cancer. Drawing on landmark clinical trials and foundational molecular studies, we highlight how these biomarkers capture spatial and temporal tumor heterogeneity in ways that conventional tissue biopsy cannot. Finally, we address the technical, clinical, and ethical challenges that still impede widespread adoption of liquid biopsy, and we outline a forward-looking research agenda oriented toward multi-omics integration and point-of-care diagnostics. Taken together, this review underscores the potential of liquid biopsy to complement existing diagnostic standards and improve how we screen for, monitor, and treat breast cancer across disease stages.

## Introduction

Breast cancer is the most frequently diagnosed cancer among women globally, and the second leading cause of cancer-related mortality [1]. According to statistics from the World Health Organization (WHO), approximately 2.3 million new cases and nearly 685,000 deaths occurred due to breast cancer in 2020 alone, representing a growing burden in both developed and low-income nations [2]. With the advent of earlier screening and more effective treatment options, many patients are now benefiting, although these services are not yet accessible to everyone. Moreover, breast cancer treatment remains particularly challenging due to the scarcity of effective interventions and the poor clinical prognosis for metastatic or treatment-resistant patients [3, 4].

Tissue biopsy is the gold standard method of oncological diagnosis since the twentieth century [5]. It is an invasive procedure that is associated with procedural risks, including bleeding, infection, and patient discomfort. Although tissue biopsy can capture only a spatially restricted snapshot of the tumor microenvironment, it may fail to represent the holistic landscape of intertumoral heterogeneity of the metastatic site of cancer [6]. Consequently, tracking the molecular pathways that drive tumor evolution, mutation, and resistance remains a significant clinical challenge due to spatial and temporal heterogeneity, though emerging technologies such as liquid biopsies and multi-region sequencing are beginning to address this limitation [7].

**Figure 1:**
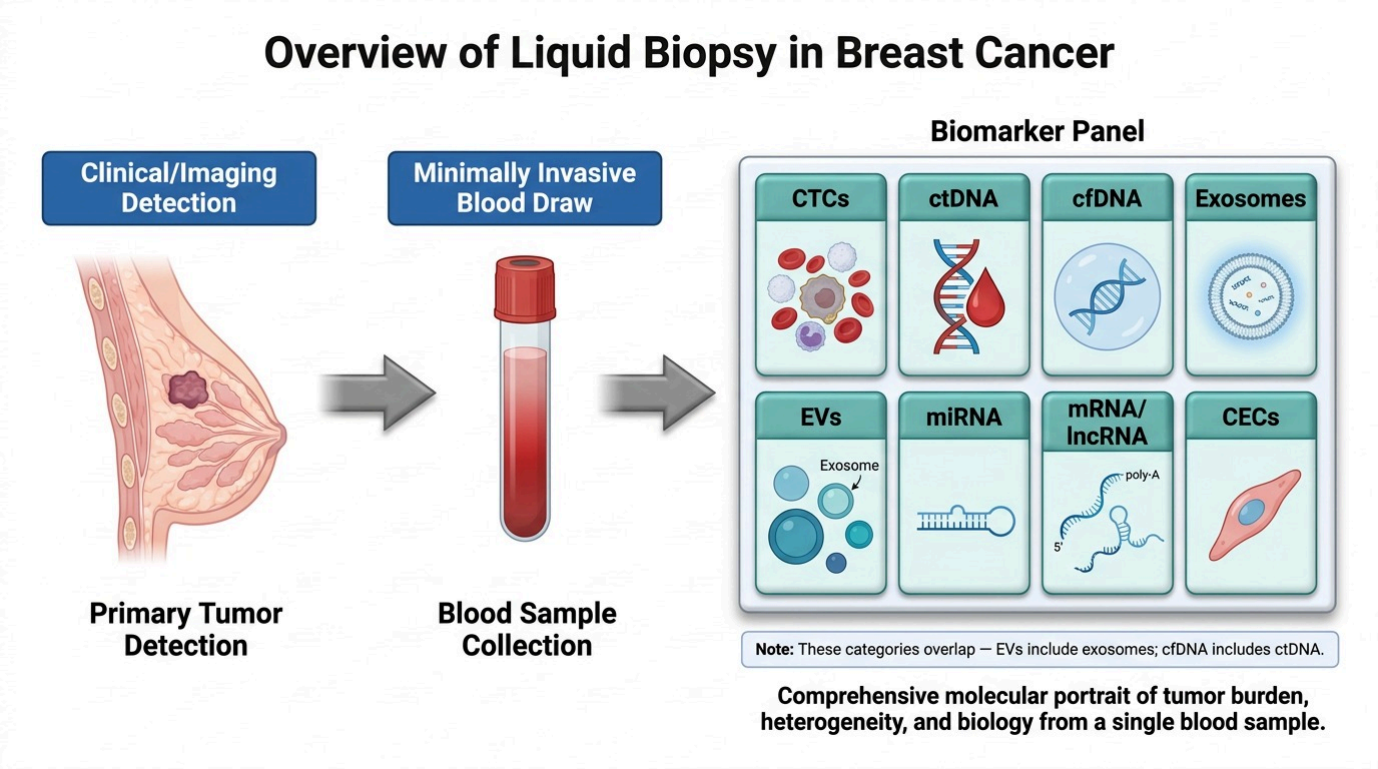
**Overview of liquid biopsy in breast cancer detection and analysis.** Schematic illustration of the liquid biopsy workflow for breast cancer. (1) Detection of breast cancer by clinical or imaging assessment identifies patients for minimally invasive sampling. (2) Liquid biopsy is performed via peripheral blood draw, capturing circulating biomarkers shed by the primary tumor into the vasculature. (3) The collected blood sample is processed and analyzed for distinct biomarker classes. It should be noted that these biomarker categories are not entirely distinct: EVs include exosomes as a subclass, and cfDNA encompasses ctDNA as a tumor-derived fraction. Together, these analytes provide a comprehensive molecular portrait of tumor burden, heterogeneity, and biology from a single blood sample. CECs: circulating endothelial cells; cfDNA: cell-free DNA; ctDNA: circulating tumor DNA; CTCs: circulating tumor cells; EVs: extracellular vesicles; miRNA: microRNA; lncRNA: long non-coding RNA.

To address unmet needs, liquid biopsy offers a dynamic and minimally invasive window into the tumor’s evolving landscape, establishing it as one of the most compelling frontiers in translational oncology [8]. Moreover, in tumor diagnosis, liquid biopsy enables sampling and analysis of biological specimens, including blood, urine, saliva, cerebrospinal fluid, and pleural effusions, to detect and characterize tumor-derived components in circulation [9]. These tumor-associated components most commonly include cell-free circulating tumor DNA (ctDNA), circulating tumor cells (CTCs), small non-coding RNA molecules such as microRNAs (miRNAs), and extracellular vesicles (EVs), which reflect the characteristics of cancer cells (Figure 1) [10, 11]. Recently, significant advancements in electrochemical biosensors, microfluidic lab-on-chip devices, surface plasmon resonance systems, and nanomaterial-based optical platforms have improved sensitivity and selectivity in cancer detection and treatment prognosis [12–14]. In addition, these advances have positioned liquid biopsy as a viable component of clinical oncology practice in low-resource settings where access to sophisticated molecular diagnostics is currently unavailable [15].

The present review aims to synthesize the current state of the liquid biopsy technique as applied to breast cancer, with a specific focus on early detection and treatment prognosis. Followed by examining the conceptual framework of liquid biopsy, we will validate the translational utility of these approaches and conclude with an appraisal of the unresolved challenges consisting of technical, clinical, and ethical perspectives, which should be navigated for widespread clinical implementation.

## Liquid biopsy: concepts, mechanisms, and clinical perspectives

The term “liquid biopsy” refers to a heterogeneous group of analytes and analytical methodologies that vary considerably in their clinical relevance, biological sources, and technical requirements for biomarker identification and diagnostic application. As a minimally invasive procedure, liquid biopsy unifies diverse analytes under a common umbrella to obtain tumor-relevant biological information from a non-solid specimen [16, 17]. It is based on the principle that tumors continuously release cells and molecular material into the bloodstream as they grow and evolve. These circulating components, derived from both primary and metastatic sites, provide a minimally invasive and real-time snapshot of tumor biology. Because their composition changes with disease progression and treatment, liquid biopsy offers a dynamic approach for monitoring cancer and therapeutic response.

The minimally invasive nature of liquid biopsy has expanded the contexts in which tumor molecular profiling can be performed, enabling serial sampling and broader clinical accessibility [18]. Traditional tissue biopsies, particularly in metastatic disease, often require image-guided procedures that involve procedural risks, patient discomfort, and logistical complexity. As a result, they are typically carried out only at diagnosis or when there is clear evidence of disease progression [19]. Consequently, the molecular information guiding clinical decision-making may be outdated and may not reflect the dynamic changes that occur as tumors evolve under therapeutic pressure [20]. In breast cancer, therapies such as aromatase inhibitors and CDK4/6 inhibitors often lead to the emergence of resistant subclones driven by newly acquired mutations [21]. Among these, alterations in the *ESR1* gene, which encodes the estrogen receptor, are especially well recognized [22]. Notably, such resistance-associated mutations can frequently be detected in ctDNA weeks before radiographic progression is evident [23]. This temporal advantage highlights the potential of serial liquid biopsy monitoring to identify molecular signs of resistance at an early stage, enabling more timely therapeutic adjustments rather than waiting for clinical or imaging-based evidence of progression.

Despite its conceptual elegance, the clinical utility of liquid biopsy in early-stage disease is frequently compromised by a signal-to-noise deficit. In patients with localized, low-volume tumors, the stochastic presence of ctDNA and CTCs often falls below the analytical sensitivity of current next-generation sequencing (NGS)-based assays, as these tumor-derived analytes are heavily diluted within the systemic circulation [24–26]. In metastatic breast cancer, ctDNA and CTCs are often present at readily detectable concentrations. In early-stage disease, these analytes are so diluted within the systemic circulation that their detection demands far more sensitive analytical platforms [27–29]. Although analytical sensitivities have been sharpened by sophisticated techniques such as NGS and droplet digital PCR (ddPCR), the diagnosis of early-stage breast cancer remains hindered by frequent false-negative results. To bridge this gap, EV-derived multi-miRNA panels are being explored as promising diagnostic biomarkers for early-stage breast cancer, complementing other liquid biopsy analytes such as ctDNA [30]. For example, combining ctDNA mutation detection with miRNA expression profiling (e.g., miR-21 and miR-155 panels) and EV surface protein markers (e.g., EpCAM-positive EVs) has demonstrated improved sensitivity for early-stage breast cancer detection compared to any single analyte alone [30]. This multi-analyte approach is particularly valuable in subtypes such as triple-negative breast cancer (TNBC) and human epidermal growth factor receptor 2 (HER2)-positive breast cancer, which differ substantially in their molecular profiles and shedding patterns. TNBC tends to shed higher levels of ctDNA due to its aggressive proliferation and genomic instability, whereas HER2-positive tumors may be better detected through protein-based markers and EV profiling [31].

In clinical applications, liquid biopsy serves as a continuous molecular “navigation system” throughout the course of breast cancer management, complementing the gaps left by traditional imaging and invasive tissue sampling [31, 32]. By characterizing the tumor’s genetic profile from a simple blood draw, it enables earlier detection in difficult-to-image dense breast tissue and identifies actionable mutations, such as *PIK3CA* or *ESR1*, to guide personalized therapy [33, 34].

**Figure 2:**
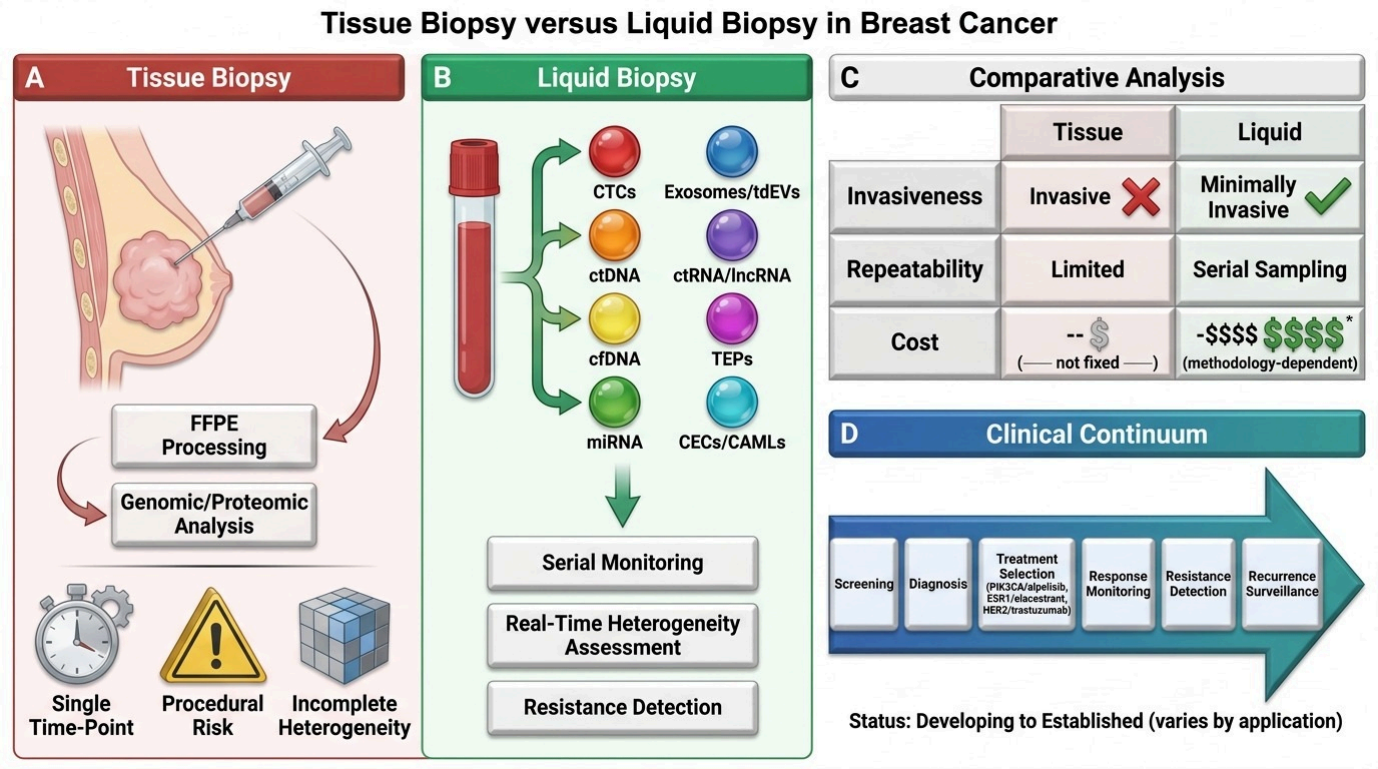
**Tissue biopsy versus liquid biopsy in breast cancer: a comprehensive comparison.** (**A**) Tissue biopsy requires invasive tumor extraction and formalin-fixed, paraffin-embedded (FFPE) processing for genomic and proteomic analysis, which is limited by single-time-point sampling, procedural risk, and incomplete capture of intratumoral heterogeneity. (**B**) Liquid biopsy enables minimally invasive, serial blood-based detection of multiple circulating analytes, including CTCs, ctDNA, cfDNA, miRNA, exosomes/tdEVs, ctRNA/lncRNA, tumor-educated platelets, and CECs/cancer-associated macrophage-like cells, supporting real-time tumor heterogeneity assessment, therapy monitoring, and resistance detection. (**C**) Comparative analysis demonstrates the advantages of liquid biopsy in terms of invasiveness, repeatability, and heterogeneity capture. Cost comparison is presented as relative ranges ($ to $$$$) rather than fixed values, as liquid biopsy costs vary considerably depending on methodology and may be comparable to or exceed tissue biopsy costs in some settings. The asterisk (*) in the original figure denotes that cost estimates are approximate and methodology dependent. (**D**) Liquid biopsy biomarkers span the breast cancer care continuum from screening and diagnosis through biomarker-guided treatment selection (e.g., *PIK3CA*/alpelisib, *ESR1*/elacestrant, *HER2*/trastuzumab), response monitoring, resistance detection, and recurrence surveillance. CECs: circulating endothelial cells; cfDNA: cell-free DNA; ctDNA: circulating tumor DNA; CTCs: circulating tumor cells; miRNA: microRNA; lncRNA: long non-coding RNA; tdEVs: tumor-derived extracellular vesicles; TEP: Tumor-educated platelet.

Beyond initial diagnosis, the real-time tracking of ctDNA levels allows clinicians to monitor treatment response months before a physical tumor appears on a scan [35]. Most significantly, detecting residual molecular disease after surgery provides a critical window for intervention, potentially preventing recurrence before it becomes clinically established. Figure 2 provides a comparative overview of liquid biopsy and tissue biopsy approaches.

It is important to emphasize that liquid biopsy should be viewed as a complement to, rather than a replacement for, conventional histopathology. Analysis of resected tumor tissue allows for a comprehensive assessment of tumor architecture, morphology, grade, invasive versus in situ components, lymphovascular invasion, receptor status, and spatial heterogeneity. These aspects remain a major advantage of tissue-based pathology and cannot currently be replaced by most liquid biopsy approaches. While liquid biopsy excels at capturing temporal molecular changes and monitoring treatment response, tissue biopsy remains indispensable for initial diagnosis, histological classification, and comprehensive tumor characterization. The integration of both modalities offers the most complete picture of tumor biology.

## Markers of interest: molecular identity and biological information

### ctDNA: the genetic fingerprint

ctDNA enters the bloodstream as a derivative of tumor cell death, incorporating the precise genetic hallmarks of the malignancy [36]. These fragments, typically 90 to 150 base pairs (bp) long, serve as a molecular lens into the tumor’s evolving genome, revealing critical mutations such as *PIK3CA* in early disease or acquired *ESR1* resistance in patients undergoing hormone therapy [37]. Beyond simple point mutations, advanced sequencing can now map methylation patterns that reveal the tumor’s tissue of origin [38]. This molecular profiling is transformative because it captures a broader genomic landscape of the cancer, complementing the limitations of traditional, localized tissue biopsies. In breast cancer subtypes, ctDNA mutation profiles differ notably: *PIK3CA* mutations are frequently observed in hormone receptor-positive (HR+) tumors, whereas TP53 mutations are more prevalent in TNBC [39].

### miRNAs: stable regulatory signatures

Unlike DNA fragments, miRNAs are small regulatory molecules that remain remarkably stable in circulation within protective protein complexes or lipid droplets [40]. This stability makes circulating miRNAs highly reliable biomarkers, and their detection at the point of care has recently become feasible through novel device platforms [41, 42]. Because miRNA levels can be elevated even when tumor DNA is scarce, they offer value in early-stage detection. MiRNA panels, such as miR-21 and miR-155, which are consistently upregulated in breast cancer, act as sensitive indicators for diagnosis [43]. Conversely, miR-205 and miR-145 are frequently downregulated in breast cancer tissues and circulation, and their reduced levels have been associated with tumor progression and poor prognosis. By shifting focus from individual molecules to integrated combinatorial panels, researchers can achieve diagnostic accuracy that far exceeds what any single marker could provide for improved detection of neoplasia [44–46].

### EVs: cellular messengers

EVs, particularly exosomes, act as carriers released by living tumor cells [47]. These membrane-bound nanoparticles protect a diverse cargo of proteins and nucleic acids from the harsh environment of the bloodstream, providing a high-reliability snapshot of the tumor microenvironment [48]. Because exosomes are actively secreted by viable cells, unlike ctDNA, which relies on cell death, they are uniquely positioned to detect early-stage tumors that may not shed substantial amounts of DNA. By analyzing the surface proteins and genetic material within these vesicles, clinicians can gain a multidimensional view of the tumor’s signaling pathways and immune evasion strategies [49]. In breast cancer, tumor-derived EVs carry elevated levels of HER2 protein in HER2-positive subtypes and display distinct miRNA cargo profiles in TNBC compared to luminal subtypes, offering subtype-specific diagnostic information [50, 51].

### Protein biomarkers and CTCs

Modern liquid biopsy platforms also target specific oncoproteins and CTCs [52]. CTCs are both unique and powerful because they are intact, viable cancer cells captured in transit through the bloodstream, offering direct insight into the disease’s metastatic potential [53]. Beyond simply counting these cells, current technologies allow for single-cell analysis to identify drug targets or resistance markers in real time. This “living biopsy” provides biological context, including protein–protein interactions and cellular heterogeneity, that molecular fragments alone cannot capture [54]. Protein biomarkers such as CA 15-3 and carcinoembryonic antigen (CEA), while limited in sensitivity when used alone, can provide supplementary value when integrated into multi-analyte liquid biopsy panels [55].

## Clinical evidence: key studies in breast cancer liquid biopsy

Liquid biopsy has emerged as an impactful approach in breast cancer diagnosis and real-time therapeutic monitoring [56]. This technique offers advantages over conventional cross-sectional imaging by enabling clinicians to track tumor evolution longitudinally, identify resistance mechanisms, assess early treatment response, and detect minimal residual disease at a stage when therapeutic intervention is most likely to be clinically impactful. Liquid biopsy has reduced reliance on surgical biopsies by analyzing ctDNA, CTCs, and cell-free RNA in the blood. The following discussion presents several significant studies demonstrating the clinical significance of liquid biopsy in breast cancer, with a critical appraisal of their strengths and limitations.

### Breast cancer diagnosis

To evaluate novel agents in the neoadjuvant setting for early high-risk breast cancer, the I-SPY 2 trial (Investigation of Serial Studies to Predict Your Therapeutic Response with Imaging and Molecular Analysis; *n* = 283 patients across multiple adaptive cohorts) utilized ctDNA profiling in TNBC and HER2-positive patients. While a single-site tissue biopsy captures only a localized snapshot of tumor biology, ctDNA profiling provides a more comprehensive genetic picture of the tumor and a representative view of the overall mutational landscape, enabling better risk stratification and improved diagnosis [57]. However, the trial’s adaptive design, while innovative, introduces complexity in interpretation, and the ctDNA findings represent a subset of the overall patient population, limiting generalizability to broader screening contexts.

The multi-cancer early detection (MCED) study analyzed genome-wide DNA methylation patterns in circulating cell-free DNA (cfDNA) to distinguish breast cancer patients from healthy controls. The test demonstrated promising diagnostic performance, with high specificity and stage-dependent sensitivity in a multi-cancer detection cohort that included breast cancer [38]. DNA methylation-based liquid biopsy is advantageous over conventional methods because cfDNA methylation signatures provide highly abundant, tissue-specific signals that enable sensitive cancer detection across diverse tumor types and stages [38]. Nevertheless, the study’s case–control design may overestimate real-world performance, and the positive predictive value in a true screening population with low disease prevalence remains to be established through prospective randomized trials [38].

Analyzing cfDNA fragmentation patterns (the fragmentome), the DNA Evaluation of Fragments for Early Interception (DELFI) study (*n* = 236 cancer patients, including breast cancer, and 245 healthy controls) introduced a novel liquid biopsy approach to identify cancer. DELFI scores were significantly elevated in breast cancer patients because cancer cells release DNA fragments with distinct size distributions compared to normal cells. Importantly, this approach requires only low-pass whole-genome sequencing, which reduces sequencing costs relative to deep targeted or whole-exome approaches [58]. A limitation of this approach, however, is that fragmentation patterns are not cancer-specific and may be influenced by non-malignant conditions such as inflammation, potentially leading to false-positive results [58].

Another significant approach is the CTC-based liquid biopsy and its prognostic value in early breast cancer detection. Generally, ctDNA and methylation-based assays struggle to detect ductal carcinoma in situ (DCIS, stage 0) because minimal tumor-derived DNA is shed at this stage. However, CTC-based approaches have shown potential as transformative tools for population-level breast cancer screening at the earliest biological stage [59]. The limited sensitivity of CTC detection in very early-stage disease, however, means that these assays are not yet suitable as standalone screening tools and should be considered as part of a multi-modal diagnostic strategy.

To assess HER2 status, a study comparing ddPCR-based liquid biopsy with standard immunohistochemistry/fluorescence in situ hybridization (IHC/FISH) in invasive ductal carcinoma identified HER2-positive status in 40% of tissue-discordant cases [60]. Unlike conventional tissue biopsy, which may not capture full tumor heterogeneity, liquid biopsy enables serial, multi-site tumor sampling and proposes a more representative HER2 profile, which can influence treatment decisions. The relatively small sample size of this study, however, warrants validation in larger prospective cohorts before routine clinical adoption.

### Breast cancer monitoring and follow-up

The PADA-1 trial (Palbociclib and ctDNA for ESR1 Mutation Detection; *n* = 1,017 HR+/HER2-negative metastatic breast cancer patients) represents one of the most compelling pieces of evidence for ctDNA-guided therapeutic decision-making to date. This trial included serial plasma ctDNA monitoring for *ESR1* mutations (a mechanism of acquired resistance to estrogen deprivation therapy) in patients receiving first-line therapy combining an aromatase inhibitor and palbociclib. Patients who were detected with emerging *ESR1* mutations were randomized either to remain on the same treatment or to switch to fulvestrant plus palbociclib. Progression-free survival was significantly improved for patients who switched therapy compared to those who remained in the original regimen. This study shows that ctDNA-based detection of *ESR1* mutations can identify patients likely to benefit from targeted therapy, supporting a broader shift toward molecularly guided treatment in advanced breast cancer [61]. A limitation of the trial is that it was conducted in a specialized oncology setting, and the infrastructure required for serial ctDNA monitoring may not be available in all clinical environments.

The SERENA-6 trial (NCT04964934; *n* = 315 HR+/HER2-negative advanced breast cancer patients) extended the paradigm established by the PADA-1 trial by introducing camizestrant, a next-generation oral selective estrogen receptor degrader. This drug was evaluated in patients who developed an emergent *ESR1* mutation detectable in ctDNA during first-line CDK4/6 inhibitor-based therapy. Patients were randomized to either continue aromatase inhibitor therapy or switch to camizestrant (75 mg once daily), both in combination with a CDK4/6 inhibitor. Progression-free survival was higher in the camizestrant group than in the aromatase inhibitor-treated group, indicating that ctDNA monitoring can detect the molecular resistance window and enable more timely disease management, which is not feasible with conventional imaging [62]. As with PADA-1, the applicability of this approach in resource-limited settings requires further evaluation.

For patients with HR+/HER2-negative metastatic breast cancer, the CTC count from a blood sample offers an alternative strategy for choosing between chemotherapy and hormone therapy as first-line treatment. The STIC CTC METABREAST trial (NCT01710605; *n* = 755 patients) incorporated this approach into a randomized phase 3 trial in which patients with ≥ 5 CTCs per 7.5 mL of blood received chemotherapy, and those with fewer CTCs received hormone therapy. Notably, despite some patients having high CTC counts, their physicians would have chosen hormone therapy based on clinical assessment alone. Using the CTC-guided approach, these patients were correctly directed to chemotherapy, and progression-free survival was improved. This highlights that clinical assessment alone may sometimes misclassify patients, and a simple blood test to detect aggressive disease can guide more appropriate treatment [63]. It should be noted, however, that the trial did not demonstrate a statistically significant improvement in overall survival, and the cost-effectiveness of routine CTC monitoring in this setting warrants further study

## Conclusions

Liquid biopsy provides a minimally invasive approach for real-time molecular monitoring in breast cancer management. While conventional tissue biopsy offers a spatially and temporally controlled view of tumor biology, liquid biopsy provides a complementary and dynamic perspective on heterogeneous tumors by assessing tumor-derived analytes, including CTCs, ctDNA, miRNAs, and EVs.

Several clinical trials, including PADA-1 and SERENA-6, have demonstrated that ctDNA-guided therapeutic strategies can significantly improve progression-free survival in HR+/HER2-negative metastatic breast cancer. In early-stage and pre-invasive disease settings, where ctDNA-based methods have limited sensitivity, CTC-based approaches such as those evaluated in the STIC CTC METABREAST trial offer an alternative detection strategy. Nevertheless, no single biomarker is sufficient for precise diagnosis. Combinatorial approaches incorporating miRNA panels, EV profiling, and integrated multi-analyte strategies represent the most promising path forward for accurate disease assessment.

Several challenges remain before liquid biopsy can achieve widespread clinical adoption. Analytical sensitivity continues to limit detection in early-stage disease, and the lack of standardized pre-analytical and bioinformatic protocols leads to variability across studies and hinders regulatory acceptance. Ambiguous findings can raise ethical concerns regarding overdiagnosis and unnecessary interventions. Furthermore, unequal access to advanced diagnostic technologies threatens to exacerbate existing health disparities. Addressing these challenges will require investment in standardized protocols, robust clinical trial infrastructure, and efforts to make these technologies accessible to patients globally. Liquid biopsy should be viewed as a powerful complement to established diagnostic standards, and its integration into routine clinical practice will depend on resolving these technical, clinical, and ethical hurdles.

## Abbreviations

cfDNA: cell-free DNA
CTCs: circulating tumor cells
ctDNA: circulating tumor DNA
ddPCR: droplet digital PCR
DELFI: DNA Evaluation of Fragments for Early Interception
EVs: extracellular vesicles
HER2: human epidermal growth factor receptor 2
HGF: hepatocyte growth factor
HR+: hormone receptor-positive
NGS: next-generation sequencing
TNBC: triple-negative breast cancer

## Declarations

### Copyright

© The Author(s) 2026.
